# Predicting protein-ATP binding sites from primary sequence through fusing bi-profile sampling of multi-view features

**DOI:** 10.1186/1471-2105-13-118

**Published:** 2012-05-31

**Authors:** Ya-Nan Zhang, Dong-Jun Yu, Shu-Sen Li, Yong-Xian Fan, Yan Huang, Hong-Bin Shen

**Affiliations:** 1 Department of Automation, Shanghai Jiao Tong University, and Key Laboratory of System Control and Information Processing, Ministry of Education of China, Shanghai 200240, China; 2 School of Computer Science, Nanjing University of Science and Technology, 200 Xiaolingwei Road, Nanjing 210094, China; 3 National Laboratory for Infrared Physics, Shanghai Institute of Technical Physics, Chinese Academy of Science, Shanghai 200083, China

**Keywords:** Protein-ATP binding site prediction, Position specific position matrix, Bi-profile sampling, Cross-validation

## Abstract

**Background:**

Adenosine-5′-triphosphate (ATP) is one of multifunctional nucleotides and plays an important role in cell biology as a coenzyme interacting with proteins. Revealing the binding sites between protein and ATP is significantly important to understand the functionality of the proteins and the mechanisms of protein-ATP complex.

**Results:**

In this paper, we propose a novel framework for predicting the proteins’ functional residues, through which they can bind with ATP molecules. The new prediction protocol is achieved by combination of sequence evolutional information and bi-profile sampling of multi-view sequential features and the sequence derived structural features. The hypothesis for this strategy is single-view feature can only represent partial target’s knowledge and multiple sources of descriptors can be complementary.

**Conclusions:**

Prediction performances evaluated by both 5-fold and leave-one-out jackknife cross-validation tests on two benchmark datasets consisting of 168 and 227 non-homologous ATP binding proteins respectively demonstrate the efficacy of the proposed protocol. Our experimental results also reveal that the residue structural characteristics of real protein-ATP binding sites are significant different from those normal ones, for example the binding residues do not show high solvent accessibility propensities, and the bindings prefer to occur at the conjoint points between different secondary structure segments. Furthermore, results also show that performance is affected by the imbalanced training datasets by testing multiple ratios between positive and negative samples in the experiments. Increasing the dataset scale is also demonstrated useful for improving the prediction performances.

## Background

Annotation of protein functions is one of the challenging tasks in bioinformatics field when facing the mass of the protein sequence data in the post-genomic Era [1-5]. It has been generally acknowledged that protein function annotation not only promotes progress of cell biology, but also benefits the development of pharmaceutical industry. However, the current situation is that there is a huge gap between large available protein sequence data and less identification of protein function. So there is an urgent desire to bridge this gap through developing accurate automated bioinformatics approaches since the wet-lab experiments are particularly laborious and expensive. In many cases, protein realizes its own specific function through interaction with other molecules or ligands in the living cell [6]. Specifically, protein is activated by its amino acid residues interacting with other residues from other proteins or small molecules and thus forms the so-called interaction interfaces [7-9]. Hence, in order to reveal the protein’s complex functions, the first critical thing is often to accurately identify these interacting residues from hundreds or even thousands of other residues. Based on these important targets, we can then recognize the protein functions either through wet-lab analysis or other dry-lab experiments.

A lot of excellent works have been reported to distinguish interacting functional residues and analyze the characteristics of interaction interface [10-13]. The early approach is based on multiple orthologous protein sequence alignment, and then assigning the most highly conserved ones as interacting residues [14-16]. Although the alignment based algorithm is demonstrated successful when a great number of protein sequence candidates is available and the aligned sequences from distinct species share similar functions [17,18], it often yields high false positive rates in the cases except for the above situations. There is hence a high desire to develop more robust methods [19,20]. Later on, analysis coupled with protein structural data has been demonstrated to be capable of improving the prediction performance than those obtained only by protein sequence features [18], where the corresponding network is modeled by the 3D structural data of the corresponding protein molecule. There is an assumption that interacting residues should be easily recognized if they have distinguishing features [21]. Based on this, many researchers begin to focus on analyzing unique sequential characteristics of interacting residues and the corresponding interaction geometry interfaces [7,22]. Nooren et al. [23] studied the composition of interacting residues and revealed existing different tendency between those residues from different type of protein complexes. It is suggested that amino acid physicochemical properties may also promote the prediction of binding residues [24-27]. Furthermore, many structural characteristics related to identification of critical residues have been intensively investigated, such as the secondary structure information etc. [28-34]. David et al. [7] has made a comprehensive characterization of protein interaction interfaces, and found that main-chain atoms contribute significantly to protein-protein interactions, where the type of interaction is highly dependent on the secondary structure type. The recently proposed DoGSite method investigated the concept of subpockets and the difference of Gaussian approach (DoG) is found to be able to improve the prediction rates of protein active sites [35]. Except for the efforts to find the discriminative features of interaction sites and their local sequential or structural environment, some studies are aiming to develop much more robust machine learning algorithms. In the report by Sankararaman et al. [36], both sequence conservation features and the structure information are integrated into a logistic regression model for a rough prediction. And then the regularized maximum likelihood approach is further exploited in the process of estimating the related parameters for avoiding overfitting phenomenon. These results show that a proper post-processing framework is helpful for yielding better prediction performance.

In this paper, we focused on the specific protein-ATP binding sites prediction, which will play essential role in reavling the mechanisms of protein-ATP complex. ATP is one kind of multifunctional nucleotides in nature composed of adenosine diphosphate (ADP) or adenosine monophosphate (AMP), acting as a coenzyme in transferring chemical energy between different cells and further supplying energy for metabolism and chemical synthesis within cells [37]. Tremendous experimental studies have shown that ATP converts itself into ADP or AMP when it releases energy previously carried, and on the contrary, ADP or AMP is transformed into ATP after it absorbed the chemical energy [38]. So ATP is constantly circulated in organisms and consumed as much as the weight of the human body each day. Naturally, ATP needs to interact with many proteins in the body to accomplish its tasks, which make it necessary to investigate ATP-protein binding residues. Of course, such knowledge can be obtained by conducting various wet-lab experiments, it can be very expensive and large time-consuming. Consequently developing computational methods for protein-ATP binding residues prediction has a great potential application. Despite the importance of the relevant research, little work was reported in this regard. Raghava et al.[39] has done some significant work on protein-ATP binding sites prediction from the amino acid sequence. They firstly constructed benchmark dataset consisting of 168 non-redundant ATP binding protein chains. Subsequently they trained several models based on different feature groups respectively such as amino acid composition of protein sequence, protein evolutionary information in the form of position specific scoring matrix (PSSM) profile, and seven physicochemical properties of amino acid. Studies by Raghava et.al revealed that evolutionary information was critical for distinguishing protein-ATP binding residues from conventional residues of protein sequence. Kurgan et.al developed a sequence based predictor called ATPsite for predicting protein-ATP binding sites and achieved very promising results [40]. In this paper, we followed these pioneer studies aiming to discover novel discriminative features around protein-ATP interactions sites and further improve the prediction performance of protein-ATP binding sites. Considering the importance of protein structural features in predicting protein functional sites in other studies, other than only the amino acid sequential features, we investigated other sequence derived structural features, i.e., protein secondary structure, protein amino acid disorder information, as well as solvent accessibility of amino acids. Furthermore, we also made use of the so-called bi-profile sampling method [41] to process these multi-view features. Our experimental results based on both the 5-fold and leave-one-out jackknife cross-validation tests show that a proper fusion of multi-view features is helpful for improving predictions of the protein-ATP binding sites.

## Methods

### Benchmark dataset

In the present study, two benchmark datasets were adopted. Firstly, we exploited the same dataset consisting of 168 protein sequences, denoted as ATP168, which was firstly organized by Raghava et al. [39]. This dataset was selected from SuperSite encyclopedia [42] and then further reduced sequence identity below 40% [43]. To further demonstrate the effectiveness of the proposed method, the second most recent dataset that contains 227 sequences constructed by Kurgan et.al [44], denoted as ATP227, was also exploited. The sequence identity of any two proteins is also less than 40% in ATP227, which is available at http://biomine.ece.ualberta.ca/ATPsite/.

In following experiments, we performed both 5-fold and leave-one-out cross-validations (LOOCV) on the ATP168 and ATP227 datasets. Taking ATP168 as an example, in the case of LOOCV, each ATP binding protein chain in the dataset was singled out in turn as the testing sample and the remaining 167 sequences constituting of the training dataset. This practice continued until all the protein chains in dataset were traversed over. Binding and non-binding residues in the training dataset were regarded as positive and negative samples to input into the machine learning models for training and prediction purposes. It should be pointed out that when constructing the positive and negative training subsets, imbalance phenomena is observed since the number of non-binding residues is far more than the number of binding residues. Our following experimental results based on selecting different ratios between positive and negative will show that the imbalance will affect the final performance of prediction model. At the beginning of the experiment design, we performed a random selection process from all non-binding residues to obtain a negative subset with the same scale as the positive dataset.

### Sequential feature extraction

The first sequential features we exploited is the position-specific scoring matrix (PSSM) profile of each protein chain generated by PSI-BLAST searching against non-redundant (nr) database composed of hundreds of millions of protein sequences. PSSM profile represents the probability of occurrence of each type of amino acid at the corresponding position that is inserted or deleted. PSSM profile for each protein can be represented as a matrix of N × 20 dimensions where N is the length of protein sequence and 20 dimensions mean a measure of residue conservation of 20 different standard amino acids. Based on the original PSSM scores, we further normalized each element in PSSM matrix using the following logistic function:

(1) $$ns=\frac{1}{1+e^{-s}}$$

where *s* is the original element in PSSM matrix and *n*s is the corresponding normalized element. Our following experiments will show that this normalization process is important for improving the prediction success rates by reducing the bias and noise contained in the original scores.

In order to reflect the amino acid types of the interacting residues and their local environment, we also encoded the amino acid residue by a 20-D vector of binary values of either zero or one according to the type of amino acid in the alphabetic order, e.g. Ala amino acid can be represented as (10000000000000000000), …, Tyr is represented as (00000000000000000001), and so forth. It should be pointed out that in this encoding way, very sparse feature vector containing few one and much more zeros is obtained that needs to be further processed for avoiding over-fitting problem in the learning and prediction steps.

### Derived feature extraction

In order to make fully use of protein sequence and prior knowledge, we also considered several derived protein features including: (1) secondary structure, (2) disorder information, and (3) solvent accessibility. We extracted the protein secondary structure information which are predicted by the state-of-art algorithm of PSIPRED [45], whose output file provides the possibility profile of all the three secondary structure states (helix, strand, and coil) for each residue in a protein sequence. Subsequently we constructed a series of N × 3 matrix based on the output file where N represents the length of peptide chain and 3 indicates the number of secondary structure types. In addition, we acquired natively unstructured region of protein sequence by DISOPRED2 server [46]. Since it was recognized as one of best servers for disordered regions prediction in protein sequence and gave out the probability whether each residue was disordered or not. And we also acquired solvent accessibility by SSpro program carried by the SCRATCH package [47] and the results obtained were in the form of the solvent accessibility status, that is, ‘exposed’ or ‘buried’ output for each residue in a protein sequence.

### Bi-profile Bayes feature space

Before we input the extracted multi-view information into the machine learning algorithms for prediction, it is important to project these features into a proper space so that the learning algorithms can make a more accurate classification. Bi-profile sampling method was firstly introduced by Shao et al. [41] in predicting methylation sites in proteins. This approach assumes that peptide chains from positive dataset should exhibit some difference in the level of amino acid characteristics compared to the amino acid of peptide chains from negative dataset. Consequently Shao et al. have achieved success in the improvement of prediction performance and good accuracy was also attained by Song et al. [48] later in predicting cleavage sites of caspase substrate. In this study, we will apply bi-profile sampling method to encode the sparse feature vector of amino acid and some unbalanced features between different categories. Those unbalanced features usually come from the uneven distribution of amino acid characteristics among different categories as well as the imbalance of the scale of positive dataset relative to negative dataset. The bi-profile sampling method can be briefly summarized as follows: given a peptide chain **P** with *n* residues without class label as $P=\left\{a_{1},a_{2},a_{3},\ldots ,a_{n}\right\}$, where $a_{i}\left(i=1,\ldots ,n\right)$ indicates the *i*th amino acid. Because in our study we are facing two categories classification task, i.e. identifying the real binding sites from those normal ones, we thus have extracted two classes of different peptide chains distinguished by their central amino acid. We further defined S^+^ as a set of peptide chains including only positive samples, and similarly we can obtained a negative dataset S^–^. For each element, that is, each single peptide chain in set S^+^ or S^–^, bi-profile sampling method means calculating a series of posterior probability related to obtained peptide chain set. Finally, we can use a vector of $v_{p}=\left(v_{1},v_{2},\ldots ,v_{n},v_{n+1},\ldots ,v_{2n}\right)$ to represent a collective posterior probability for one peptide chain **P**, where the first *n* elements of $\left(v_{1},v_{2},\ldots ,v_{n}\right)$denote the posterior probability of each amino acid at the corresponding position of the peptide chain **P** compared to the positive dataset S^+^, while $\left(v_{n+1},v_{n+2},\ldots ,v_{2n}\right)$ indicates the posterior probability of each amino acid at the corresponding position of the peptide chain P compared to the negative dataset S^–^.

**Figure 1:**
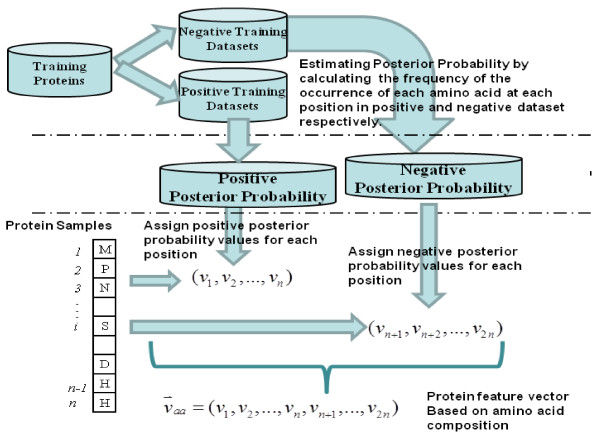
A framework diagram showing how to extract protein feature vector based on amino acid composition and bi-profile method.

We then use the above bi-profile approach to model the multi-view information. Firstly, we estimated the posterior probability by calculating the frequency of the occurrence of each amino acid at each position in positive and negative dataset respectively. Then we got the vector $v_{\mathrm{aa}}=\left(v_{1},v_{2},\ldots ,v_{n},v_{n+1},\ldots ,v_{2n}\right)$ for each peptide chain and we called v_aa_ the bi-profile sampled vector based on amino acid composition. We can further constructed bi-profile sampled vector based on protein secondary structure. Specifically, we calculated the posterior probability of three different protein secondary structure respectively at the corresponding position of each peptide chain compared to the positive dataset S^+^ as well as negative dataset S^–^, then we also obtained $v_{\mathrm{ss}}=\left(v_{1},v_{2},\ldots ,v_{n},v_{n+1},\ldots ,v_{2n}\right)$. Similarly, we further extracted bi-profile sampled vector v_*dis*_ based on residue disorder information as well as bi-profile sampled vector v_*sa*_ based on solvent accessibility of amino acid. Figure 1 shows an intuitive diagram of how to extract bi-profile feature vectors. Table 1 illustrates the discrete features that adopted in this study.

**Table 1:** Summarization of different feature types and their vector representation dimensions

**Feature types**	**Dimensionality**
Position-specific scoring matrix (PSSM)	340
Position-specific scoring matrix normalized by logistic function (LogisticPSSM)	340
Bi-profiled binary amino acid composition (Bipro-aa)	34
Bi-profiled predicted secondary structure (BiPro-ss)	34
Bi-profiled predicted residue disorder (BiPro-dis)	34
Bi-profiled predicted residue solvent accessibility (Bipro-sa)	34

### Support vector machines (SVM)

SVM is a machine learning approach based on structural risk minimization principle of statistical learning theory, which has been successfully applied in various bioinformatics researches [49-52]. In this paper, we exploited the freely available software package libsvm-3.11 developed by Chang and Lin and we also selected Radial Basis Function (RBF) as the kernel function since RBF has been demonstrated to be an optimal kernel in many cases. Then there are two parameters of capacity parameter *C* and kernel width *g* needing to optimize by a grid search approach [53]. For further theoretical details about SVM, please refer to [54].

### Evaluation criteria

The overall accuracy (Accuracy) is one of the most commonly used indexes for evaluating the performance of a classifier. This index provides a simple way of describing a classifier’s performance on a given data set. However, in the situation of imbalanced learning scenario, using overall accuracy index alone is no longer appropriate and can be deceiving for evaluating a classifier on imbalanced dataset. Thus, in this study, the overall accuracy index together with several other indexes (Specificity, Sensitivity, and Precision), are adopted to provide comprehensive assessments of the developed ATP binding sites predictor.

(2) $$Specificity=\frac{TN}{TN+FP}$$

(3) $$Sensitivity=\frac{TP}{TP+FN}$$

(4) $$Precision=\frac{TP}{TP+FP}$$

(5) $$Accuracy=\frac{TP+TN}{TP+TN+FP+FN}$$

where TP, FP, TN, and FN are abbreviations of the number of true positive, false positive, true negative, and false negative samples respectively. We also exploit Receiver Operating Characteristic (ROC) curve, which is a plot between true positive proportion (TP/TP + FN) and false positive proportion (FP/FP + TN). The area under the ROC curve (AUC), which increases in direct proportion to the prediction performance, is also calculated.

Another important issue should be addressed here is how to objectively report the evaluation indexes listed above, especially in the situation of imbalanced learning scenario. Let’s commence by considering how a ROC curve is calculated. For a soft-type classifier, i.e., classifier that output a continuous numeric value to represent the confidence of a sample belonging to the predicted class, gradually adjusting classification threshold will produce a series of confusion matrices [55]. From each confusion matrix, a ROC point, the coordinate of which is (TP/TP + FN, FP/FP + TN), can then be computed. A series of ROC points constitute the ROC curve. In other words, different ROC point corresponds to a different confusion matrix, from which the evaluation indexes, i.e., Accuracy, Specificity, Sensitivity, and Precision, can be computed. Then, based on which ROC point should we report the evaluation indexes of Eqs.(2)-(5)?

**Figure 2:**
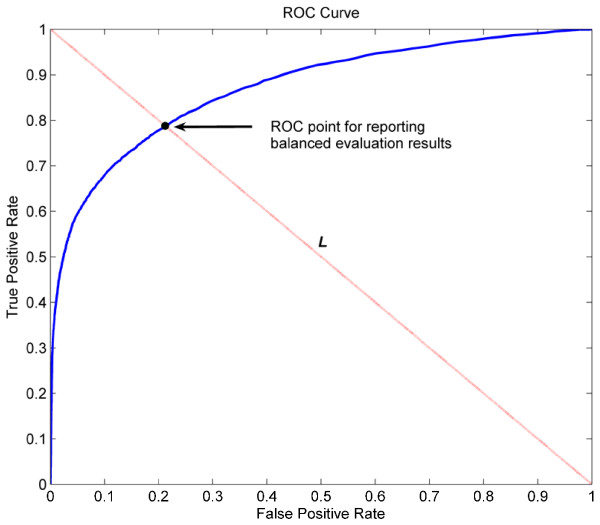
Illustration of determining the ROC point on a ROC Curve for reporting balanced evaluation results.

Considering the actual requirement of the protein-ATP binding sites prediction problem, we expect that a classifier can provide high accuracy for the minority class (binding site) without severely jeopardizing the accuracy of the majority class (non-binding site). In light of this, we believe that it is more appropriate and objective to report evaluation results for both minority and majority classes in a balanced manner. More specifically, we would rather report evaluation results based on the ROC point where the value of the false positive rate is the same as that of the false negative rate [56]. As shown in Figure 2, the ROC point for reporting balanced evaluation results in this study is the intersection point (black circle) of the ROC curve and the line *L* that passes through points (0,1) and (1,0).

## Results and discussion

### Analyzing the determinants of binding specificity

**Figure 3:**
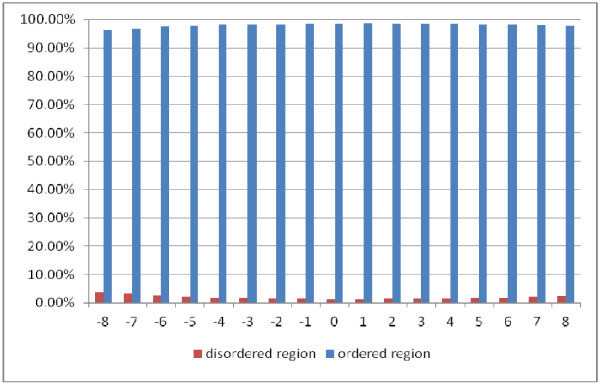
Residue disorder knowledge statistics on positive peptide chains around the binding residues on ATP168: position 0 means the binding site.

**Figure 4:**
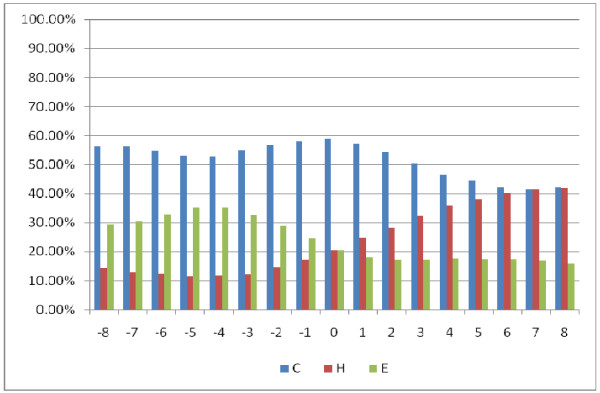
Residue predicted secondary structure (C: coil, H: helix, E: strand) knowledge statistics on positive peptide chains around the binding residues on ATP168: position 0 means the binding site.

**Figure 5:**
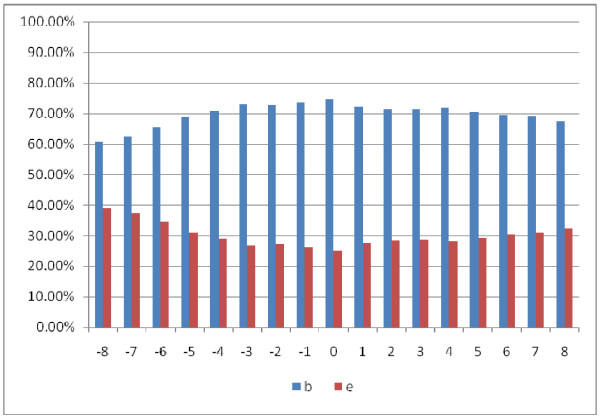
Residue predicted amino acid solvent accessibility (b: buried, e: exposed) statistics on positive peptide chains around the binding residues on ATP168: position 0 means the binding site.

**Figure 6:**
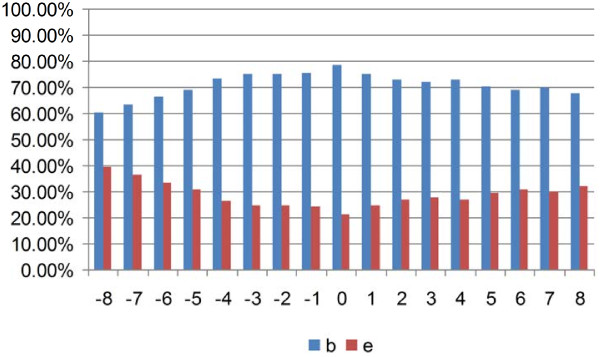
Residue predicted amino acid solvent accessibility (b: buried, e: exposed) statistics on positive peptide chains around the binding residue on ATP227.

As stated earlier, we performed the prediction of native disorder information (disordered, ordered), residue three secondary structures (helix, strand, and coil), and amino acid solvent accessibility (exposed, buried) respectively by the DISOPRED2 server [46], PSIPRED [45], as well as SCRATCH package [47]. We extracted positive peptide subset in the form of peptide chains derived from a sliding window whose central residues are binding residues and the window length is 17 residues. Figures 3,4, and 5 show the statistical information on the positive subset for the three kinds of features on the ATP168 dataset respectively. Firstly, as the native disorder information shown in Figure 3, we can see that the percentage of disordered amino acids is much smaller than those of ordered residues around the binding sites; at the same time, it has also shown that the fraction reaches the lowest at the position of the middle of peptide chains (binding site). This indicates that the protein-ATP bind regions prefer more to the structural states than the unstructural [57-59]. In Figure 4, we can see that the percentage of amino acids located in the margin is significantly different from those settled in the central region based on three types of secondary structure (helix, strand, and coil). It is interesting to find out that at the binding site (position 0), the percentage of helical residues and strand residues are almost equal; however, the fractions of helical residues keep going up on the right side, while the fractions of strand residues keep going down. This statistic data indicates that the binding residues could prefer to a conjoint point of two different secondary structural segments. Figure 5 illustrates the predicted residue solvent accessibility statistics. As indicated in Figure 5, the protein-ATP binding sites do not show significant exposed feature, on the contrary, they have higher buried propensities compared with the residues around. This finding is somewhat different from our previous knowledge that exposed residues are more easily for binding [60]. In order to re-demonstrate this finding, Figure 6 illustrates the residue solvent accessibility statistics on ATP227, which is similar to that of Figure 5.

We also analyzed the distributions of different structural features on real binding sites and those normal ones in both ATP168 and ATP227 datasets, which are summarized in Table 2. Results of Table 2 reveal that statistics on both datasets of 168 and 227 sequences are consistent and structural features of real binding residues are significantly different from those non-binding residues. For example, ATP interactions prefer to happen at ordered regions (98.0% and 97.8% on both datasets), and they in most cases are observed of buried status (75.3% and 78.7% on both datasets). The distributions on secondary structural types of real binding and non-binding residues also show differences. In ATP168, the Coil (C), the Helical (H) and the Strand (E) in binding sites account for 49.6%, 26.0% and 24.4%, respectively; while in ATP227, they account for 49.3%, 24.6% and 26.1%, respectively.

**Table 2:** Comparisons of residue structural features between real binding sites and those normal ones in ATP168 and ATP227

**Residue Structural features**	**Binding Sites**	**Non-binding Sites**
Ordered^a^	98.0%	91.9%
Disordered^a^	2.0%	8.1%
Ordered^b^	97.8%	91.9%
Disordered^b^	2.2%	8.1%
Coil (C)^a^	49.6%	41.7%
Helical (H)^a^	26.0%	40.3%
Strand (E)^a^	24.4%	18.0%
Coil (C)^b^	49.3%	41.7%
Helical (H)^b^	24.6%	40.4%
Strand (E)^b^	26.1%	17.9%
Exposed (E)^a^	24.7%	41.5%
Buried (B)^a^	75.3%	58.5%
Exposed (E)^b^	21.3%	41.5%
Buried (B)^b^	78.7%	58.5%

^a^ Statistics on ATP168 dataset.

^b^ Statistics on ATP227 dataset.

### Feature normalization is helpful for improving performance

In our experiments, we generated position-specific scoring matrix (PSSM) as evolutionary profile of each protein chain by PSI-BLAST searching against non-redundant (nr) database. By applying the sliding window with length of 17 on the original PSSM profile in the form of N × 20 matrix, we finally acquired a vector of 340 dimensions for each peptide chain from the training dataset. Then we directly performed 5 fold cross-validation in this training dataset by SVM algorithm and got average accuracies of 75.82% and 78.16% on ATP168 and ATP227 datasets respectively. Subsequently we normalized the vector of 340 dimensional vector by the logistic function of Eq.(1) and again performed 5 fold cross-validation on the same training datasets with the same SVM classifier. We acquired average accuracies of 76.60% and 79.57% on the two datasets (Table 3). Similar results have also been observed in the jackknife test (Table 4). These improved results demonstrate that a proper normalization process is helpful for reducing the bias among different protein samples and thus can yield better accuracy.

**Table 3:** Combination of different feature groups and performance comparison based on 5-fold cross-validation tests on ATP168 and ATP227

	**Composition of different features**	**Sen (%)**	**Spe (%)**	**Acc (%)**	**AUC**
ATP168	PSSM	75.67	75.71	75.82	0.8410
	LogisticPSSM^a^	76.28	76.62	76.60	0.8493
	LogisticPSSM + Bipro-aa	76.90	77.19	77.18	0.8553
	LogisticPSSM + Bipro-dis	76.64	77.09	77.07	0.8537
	LogisticPSSM + Bipro-sa	77.00	77.48	77.46	0.8562
	LogisticPSSM + Bipro-ss	77.35	77.58	77.56	0.8579
	LogisticPSSM + Bipro-all^b^	77.00	77.32	77.30	0.8569
ATP227	PSSM	78.31	78.16	78.16	0.8609
	LogisticPSSM^a^	79.17	79.59	79.57	0.8727
	LogisticPSSM + Bipro-aa	79.77	79.91	79.91	0.8770
	LogisticPSSM + Bipro-dis	79.71	79.89	79.88	0.8763
	LogisticPSSM + Bipro-sa	79.95	80.12	80.11	0.8797
	LogisticPSSM + Bipro-ss	80.07	80.31	80.30	0.8813
	LogisticPSSM + Bipro-all^b^	79.95	80.27	80.26	0.8800

^a^ LogisticPSSM means PSSM profile normalized by logistic function.

^b^ all means the combination of four feature groups: Bipro-aa, Bipro-dis, Bipro-sa, and Bipro-ss.

**Table 4:** Combination of different feature groups and performance comparison based on leave-one-out jackknife tests on ATP168 and ATP227

	**Composition of different features**	**Sen (%)**	**Spe (%)**	**Acc (%)**	**AUC**
ATP168	PSSM	76.47	76.75	76.74	0.8466
	LogisticPSSM^a^	77.22	77.55	77.54	0.8553
	LogisticPSSM + Bipro-aa	77.45	77.71	77.70	0.8609
	LogisticPSSM + Bipro-dis	77.45	77.76	77.74	0.8601
	LogisticPSSM + Bipro-sa	77.45	77.96	77.93	0.8619
	LogisticPSSM + Bipro-ss	77.84	78.20	78.18	0.8638
	LogisticPSSM + Bipro-all^b^	77.75	78.16	78.13	0.8633
ATP227	PSSM	79.23	79.63	79.61	0.8678
	LogisticPSSM^a^	79.98	80.14	80.14	0.8788
	LogisticPSSM + Bipro-aa	80.30	80.54	80.53	0.8822
	LogisticPSSM + Bipro-dis	80.36	80.53	80.53	0.8818
	LogisticPSSM + Bipro-sa	80.54	80.76	80.75	0.8843
	LogisticPSSM + Bipro-ss	80.57	80.85	80.83	0.8851
	LogisticPSSM + Bipro-all^b^	80.54	80.60	80.70	0.8816

^a^ LogisticPSSM means PSSM profile normalized by logistic function.

^b^ all means the combination of four feature groups: Bipro-aa, Bipro-dis, Bipro-sa, and Bipro-ss.

### Performance by fusing multi-view derived features

The above results have demonstrated that evolutionary profile has relatively good discrimination ability between binding residues and non-binding residues, we then try to further improve the prediction performance by incorporating other multi-view features into the model. As bi-profile sampling method has been demonstrated successful in other studies [41,48], we then applied this technique to encode the following features as discussed above: (1) binary value of amino acid composition, (2) predicted protein secondary structures, (3) predicted protein amino acid disorder information, and (4) predicted protein solvent accessibility. Because the sliding window for extracting features is 17 and based on the flowchart shown in Figure 1, we then can use a 34-D vector to represent the above 4 features, abbreviated as Bipro-aa, Bipro-ss, Bipro-dis, and Bipro-sa respectively. Table 1 summarizes all the feature information and their vector representation dimensions.

Subsequently we will combine different feature groups and compare their discrimination ability in the corresponding trained models. For reference, we firstly list performance comparisons based on 5-fold cross-validation among combinations of different feature groups in Table 3. As shown in Table 3, more or less improvements can be observed by incorporating any of four feature groups into logistic PSSM profile on both ATP168 and ATP227 datasets. Taking ATP168 as an example, when selecting LogisticPSSM profile coupled with predicted protein secondary structure information, the prediction accuracy performs best in all of compositions in different feature groups, which is 77.56% accuracy. This probably means that protein secondary structure information has better discriminative ability for classifying binding sites from the non-binding sites, which is consistent to our analysis on the Figure 4. When plotting the receiver operating characteristic (ROC) curves for the corresponding models in Table 3, we find that the AUC criterion have also been improved by fusing other information into the evolutionary features. For example, the AUC is 0.8579 in LogisticPSSM and Bipro-ss input case compared with 0.8493 in LogisticPSSM as the independent input feature. Table 4 illustrates the results obtained from the leave-one-out (jack-knife) test, and all the criterion have been improved when considering multi-view features. These results show that different features have their own merits and shortcomings, and fusion process can make them be complementary to each other. Similar results are also observed on ATP227 dataset as shown in Tables 3 and 4.

It is notable from Tables 3 and 4 that the incorporation of PSSM profile and all of the other four kinds of bi-profile features does not lead to the best prediction performance. This phenomenon indicates that not all the features that can be calculated are useful. At the same time, when we simply serially combine these features together, such combination of features will simultaneously increase the information redundancy that could, in turn, deteriorate the final accuracy. Considering of this, a proper feature fusion and selection approach should be discussed in the future in this regard to further improve the prediction performance.

### Imbalanced learning effects

**Figure 7:**
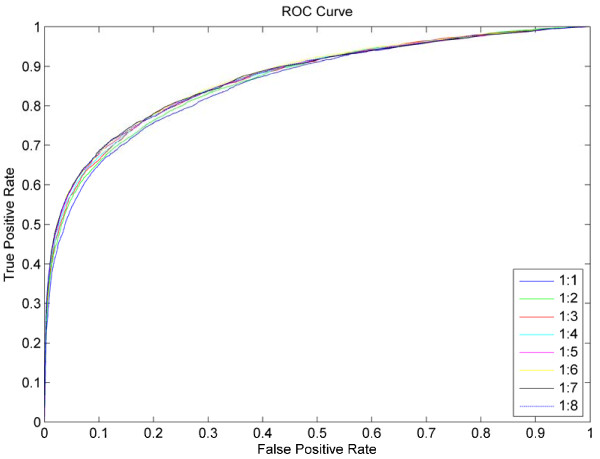
ROC curves of eight training datasets with different sizes based on 5-fold cross validation tests on ATP168. 1:1, 1:2, …, and 1:8 mean that the sizes of negative training subsets are 1 ~ 8 times respectively the sizes of corresponding positive datasets.

Since the number of non-binding residues is far more than the number of binding residues, we have performed a random selection process from all non-binding residues of each protein sequence so as to balance the negative dataset with the positive dataset in previous experiments. In order to further study the impact of the scale of negative dataset on prediction performance, we continue to randomly extract negative datasets of 2N, 3N,…, and 8N, where N is the scale of the positive dataset. The eight negative datasets plus the positive dataset from ATP168 then constitute eight new training datasets with the ratio of 1:1, 1:2, …, and 1:8 between positive and negative samples. We constructed feature vectors based on PSSM profile and Bi-profiled secondary structure on the 8 training datasets. After training the models, Figure 7 plotted corresponding ROC curves of these different size training datasets based on 5-fold cross-validation tests. The AUCs are 0.8579, 0.8639, 0.8677, 0.8684, 0.8690, 0.8720, 0.8706, and 0.8697 respectively. These results indicate that results are indeed affected by the imbalanced training dataset.

### Performance affected by dataset size

The core of statistical learning algorithms is learning prediction rules from training samples. Different sizes of the two datasets applied in this study make us capable of studying the prediction performances affected by the dataset scales. All the results listed in Tables 3 and 4 demonstrate that performances are consistently better when classifiers are trained on the ATP227 dataset compared with those on ATP168 on both 5-fold and jackknife cross validation tests. The results are typically improved by 2%–3% under the same feature inputs. For example, the AUC is 0.8638 when training model on ATP168 with LogisticPSSM and Bipro-ss as inputs in jackknife test, while this value increases to 0.8851 on ATP227. These results reveal that it is important to collect as many training samples as possible to make the learning rules more accurate. This is particularly important when studying the small-sample problems where experimentally derived knowledge is very limited in many cases.

### Comparison with other methods

We firstly compare our results with previous results obtained by Raghava et al. [39] on the same dataset of ATP168. In the previous study, the authors acquired an average accuracy of 75.11% based on 5-fold cross-validation on ATP168, while we achieved an accuracy of 77.56% by incorporating predicted protein secondary structure feature into PSSM profile (with logistic normalization) also based on 5-fold cross-validation. In the leave-one-out cross-validation test, 78.18% accuracy through combination of PSSM profile and predicted protein secondary structures is achieved. After constructing the ATP227 dataset by Kurgan lab, a predictor called ATPsite has been constructed by the same group [44]. An AUC value of 0.854 has been reported in ATPsite on ATP227 dataset. On the same dataset, the AUC values are 0.8813 and 0.8851 in 5-fold and jackknife cross-validation tests respectively when incorporating LogisticPSSM and Bipro-ss as the input features in this study. These results indicate that the performance of current study is superior to the state-of-the-art approaches, which can play important complementary roles with existing predictors. It is expected that prediction performance could be further enhanced when performing consensus predictions based on these multiple predictors.

## Conclusions

In this paper, we developed a protocol for prediction of protein-ATP binding residues. In order to reflect the multi-view characteristics of binding residues, multiple sequential and sequence derived structural features are exploited, which are further encoded by the bi-profile sampling approaches. Our experimental results show that prediction performance can be improved by fusing multi-view features. Furthermore, we show that increasing dataset size can also be helpful for enhancing the power of ATP binding residue predictors. Performances are found to be affected by the imbalances between positive and negative samples. Current prediction protocol is expected to play an important complementary role to the existing approaches for large-scale ATP binding protein function annotation.

## Competing interests

The authors declare that they have no competing interests.

## Authors’ contributions

Conception and design YNZ, DJY, YH, HBS; Acquisition of data YNZ, SSL, YXF; Analysis of data YNZ, DJY, SSL, YXF, YH, HBS; Writing and revising the manuscript: YNZ, DJY, YH, HBS. All authors read and approved the final manuscript.
